# Repeated Transarterial Radioembolization Adverse Event and Hepatotoxicity Profile in Cirrhotic Patients With Hepatocellular Carcinoma: A Single-Center Experience

**DOI:** 10.7759/cureus.23578

**Published:** 2022-03-28

**Authors:** Dustin K Reed, William H Stewart, Travis Banta, Seth T Lirette, Garth S Campbell, Akash Patel

**Affiliations:** 1 Radiology, University of Mississippi Medical Center, Jackson, USA; 2 Data Science and Radiology, University of Mississippi Medical Center, Jackson, USA

**Keywords:** radioembolization induced liver disease (reild), hepatotoxicity, adverse event profile, cirrhosis, hepatocellular carcinoma (hcc), yttrium-90, repeat transarterial radioembolization

## Abstract

Purpose

The study aimed to evaluate the adverse event (AE) and hepatotoxicity profile, including radioembolization induced liver disease (REILD), following repeat radioembolization (RE) to the same or overlapping vascular territories in patients with hepatocellular carcinoma (HCC) and limited functional hepatic reserve/cirrhosis.

Methods

Nine patients (seven male and two female; median age, 66 years) with cirrhosis and HCC who underwent repeat RE (cycle 1 and cycle 2) between January 2012 and August 2019 were included. Patient demographics, clinical and treatment history, and pertinent laboratory values were recorded at baseline and post-treatment time points over a period of four months. Post-RE AE/hepatotoxicity was assessed, organized by type and frequency, and graded by severity according to the National Cancer Institute common terminology criteria for adverse events, version 5.0 (CTCAE v5.0). To assess rudimentary comparisons for post-RE hepatotoxicity vs. factors of interest, Spearman's rank correlation/rho was calculated, and all relevant plots were constructed. Kaplan-Meier analysis was performed along with associated median survival time. All statistical analyses were performed with Stata v16.1.

Results

Following cycle 1, 22 objective AE were identified according to CTCAE v.5 (17 grade I, four grade II, and one grade III), with grade I, II, and III AE experienced by 78%, 33%, and 11% of patients, respectively. Following cycle 2, 19 objective AE were identified according to CTCAE v.5 (11 grade I, seven grade II, and one grade III), with grade I, II, and III AE experienced by 89%, 56%, and 11% of patients, respectively. A single patient developed REILD after cycle 1, which progressed to fatal REILD following cycle 2. Following cycle 2, an additional patient advanced from less severe hepatotoxicity to REILD. Following cycle 2, positive correlations between the higher model for end-stage liver disease (MELD; rho=0.70) and Child-Pugh (rho=0.74) scores and degree of post-RE hepatotoxicity/REILD appear to emerge. Post-repeat RE median overall survival was 12.5 months.

Conclusion

Post-RE hepatotoxicity following repeat RE to the same or overlapping vascular territories in patients with limited functional hepatic reserve/cirrhosis is a common occurrence with variable severity ranging from transient laboratory derangement to fatal REILD. Lack of a consensus REILD definition and grading scale results in non-uniform reporting of incidence as well as clinical and laboratory features of the disease process. Strides aimed at improving clinical characterization, forming a more complete diagnostic definition, and establishing a uniform grading system with respect to REILD are of particular importance and would ultimately improve repeat RE patient selection and risk management.

## Introduction

Over the past three decades, hepatocellular carcinoma (HCC) incidence rates have been rising, and these trends are expected to continue through 2030 [1]. Between 75% and 85% of HCC is associated with underlying cirrhosis (i.e., limited hepatic functional reserve) due to a number of underlying factors, including viral hepatitis B or C infection (HBV or HCV), alcohol abuse, auto-immune hepatitis, non-alcoholic steatohepatitis (NASH) as well as other less common etiologies. For those patients with advanced disease at presentation or recurrence following curative intent procedures, such as transplant, surgical resection, or thermal ablation, treatment is aimed at prolonging survival and/or mitigating symptoms [2]. Yttrium-90 (^90^Y) radioembolization (RE) has emerged as a viable treatment option in these cases. Given the arterially hypervascular nature of HCC, these microspheres preferentially lodge within the microvasculature in and around the tumor, where they selectively deliver remarkably high radiation doses [3,4]. Overall, a favorable toxicity profile has been seen following RE; however, some degree of liver toxicity is seen in nearly all patients treated with RE [2]. Established and not unexpected toxicities include post-RE syndrome (fatigue, fever, pain, nausea) and transient leukopenia, thrombocytopenia, and altered liver function (increased transaminase and bilirubin levels, decreased albumin serum level) [4]. Less common, though more severe adverse events (AE) following RE include non-target embolization, radiation pneumonitis, and radioembolization-induced liver disease (REILD) [5].

Though several series report REILD occurring at a rate of 0 to 8%, the defining features and natural course of REILD and lesser forms of post-RE hepatotoxicity vary greatly in the literature. [3,6-8]. Surprisingly, following an initial RE, there are relatively few data available to guide how patients should be further treated, particularly related to toxicity risk following repeat-RE. Furthermore, while multiple risk factors predisposing to post-RE hepatotoxicity and REILD have been described, there have been somewhat conflicting reports regarding a potentially increased risk of post-RE hepatotoxicity and REILD following repeat RE to the same or overlapping vascular distribution in patients with and without impaired baseline liver function [2-4,8-17]. Accordingly, there is developing discussion regarding REILD, and lesser forms of post-RE hepatotoxicity, beyond the significant work in understanding complication profiles following a single RE treatment.

This study retrospectively reviews nine patients with HCC in the setting of background cirrhosis (i.e., limited hepatic reserve and/or decreased baseline liver function) who underwent repeat RE to the same or overlapping vascular territories. The primary endpoint was to identify hepatic toxicity/REILD following repeated RE in a cohort of patients uniformly affected by HCC and cirrhosis.

## Materials and methods

Patients and study design

The local institutional review board approved this single-center, Health Insurance Portability, and Accountability Act (HIPPA)-compliant study and waived written informed consent. A retrospective review of the 188 patients undergoing RE at this institution (1/2012-8/2019) was performed. Patients included in the cohort had unresectable HCC in the setting of background cirrhosis treated with repeat RE to the same or overlapping vascular territories. A multi-disciplinary tumor board guided treatment recommendation prior to each RE treatment cycle. Patients with resectable HCC, with other forms of primary or metastatic malignancy affecting the liver, not treated with repeat RE to the same or overlapping vascular territories, and/or lacking cirrhotic liver morphology were excluded. For each patient, demographics, Barcelona Clinic Liver Cancer (BCLC) staging, and Eastern Cooperative Oncology Group (ECOG) performance status, baseline and post-treatment laboratory values, Child-Pugh (CP) and model for end-stage liver disease (MELD) scores, treatment history including locoregional, surgical, and/or systemic therapies, and RE treatment characteristics were evaluated. MELD scores were calculated according to the most recent Organ Procurement and Transplant Network (OPTN) 2016 guidelines; additionally, standard MELD exceptions were also applied [18]. Data were obtained by searching the electronic medical record (EMR) of each patient.

Radioembolization technique and treatment protocol

Pre-treatment/mapping angiography was performed to identify tumoral vascular supply with confirmation using cone-beam computed tomography (CBCT); identify and embolize any vessels that may lead to non-target deposition of radioembolic material, and quantify lung shunt fraction (LSF) and identify splanchnic or non-target flow by intra-arterial injection of Technetium-99m macroaggregated albumin (^99m^Tc-MAA) and subsequent imaging. Single-photon emission computed tomography (SPECT) and planar nuclear imaging were performed to assess extra-hepatic deposition and calculate the lung shunt fraction (LSF), respectively. Based off LSF study results, ^90^Y dose administration was planned as outlined by package insert parameters.

RE treatment cycles were performed within one month following the pre-treatment/mapping evaluation except for one patient for which only a single pre-treatment evaluation was performed due to a short interval between treatment cycles. The prescribed activity of ^90^Y resin or glass microspheres was calculated for each individual patient utilizing the body surface area method or calculated to correspond to the injected liver volume desired dose, respectively. Following the procedure, the distribution of ^90^Y-microspheres was assessed by planar bremsstrahlung imaging alone or in combination with ^90^Y -positron emission tomography-computed tomography (PET-CT) or fused SPECT.

Prior, inter-cycle or follow-up period treatments

Compiled from the EMR and available outside facility records, a comprehensive treatment history was established and documented for each patient, including any prior, inter-cycle, or follow-up period systemic, surgical, and/or locoregional therapies.

Clinical, imaging and laboratory toxicity assessment

Baseline laboratory, imaging, and clinical findings were documented prior to the cycle 1 treatment. Given the treatment interval length and multiple patients undergoing inter-cycle treatments, a second baseline was established prior to the cycle 2 treatment. Pre- and post-procedural laboratory data of interest were recorded. Given the reported variable onset of REILD ranging from two weeks to four months post-RE [3], relevant laboratory, clinical, and imaging data were reported up to four months following each treatment when available. From these data, liver toxicities and AE were graded according to the National Cancer Institute common terminology criteria for adverse events, version 5.0 (CTCAE v5.0) [19]. These data were then used to further stratify post-RE hepatotoxicity according to a grading system established by Braat et al. [3].

Statistical analysis

Medians, ranges, interquartile ranges (IQR), counts, and percentages were compiled where appropriate. To assess rudimentary comparisons of post-RE hepatotoxicity vs. factors of interest (radiation dose to the perfused liver, targeted tissue mass, baseline MELD, baseline Child-Pugh Score, and the number of prior or inter-cycle treatments), Spearman's rho was calculated, and all relevant plots were constructed. Kaplan-Meier analysis was performed along with associated median survival time. All statistical analyses were performed with Stata v16.1 (StataCorp LLC, College Station, USA).

## Results

Repeat radioembolization treatments

A cohort of nine patients meeting the above inclusion criteria was identified (Table 1). At the time of treatment cycle 1, the median patient age was 66 years (range, 50-75 years; IQR, 17). Cirrhosis etiologies included cryptogenic (n=1), HCV (n=3), alcoholic (n=3), NASH (n=1), and combined HCV and alcoholic (n=1). Baseline ECOG status was 0 in three patients and 1 in the remaining six patients. The pre-cycle 1 median baseline MELD score was 10 (range, 6-22; IQR, 15), and the median Child-Pugh score was 6(A) (range, 5(A)-7(B); IQR, 1). The baseline BCLC stage was A for one patient, B for seven patients, and C for the remaining patient.

**Table 1:** Baseline patient characteristics prior to treatment cycle 1 radioembolization HCC: hepatocellular carcinoma; HCV: hepatitis C virus; NASH: non-alcoholic steatohepatitis; ECOG PS: Eastern Cooperative Oncology Group performance status; BCLC: Barcelona Clinic liver cancer-stage; MELD: model for end-stage liver disease; IQR: interquartile range

Characteristic	Value
Age in years (at treatment cycle 1)	
median	66
range; IQR	50-75; 17
Sex, n	
Male	7
Female	2
Tumor etiology, n	
HCC (with cirrhosis)	9
Extrahepatic metastasis	0
Cirrhosis etiology, n	
Cryptogenic	1
HCV	3
Alcoholic	3
NASH	1
HCV + Alcoholic	1
Baseline ECOG PS, n	
0	3
1	6
Baseline BCLC stage, n	
A	1
B	7
C	1
Baseline MELD score, n	
≤ 9	4
> 9	5
median	10
range; IQR	6-22; 15
Baseline Child-Pugh score, n	
median	6/A
range; IQR	5/A–7/B; 1

Each patient underwent two RE treatment cycles covering the same or overlapping vascular territories in varying fashion: lobar, n=4; segmental, n=3; and mixed lobar/segmental, n=2 (Table 2). The median interval between cycles 1 and 2 was nine months (range, 2-17 months; IQR, 11.3 months). Pre-cycle 1 LSF median was 5.5% (range, 2.7-10.2%; IQR 3.0%). Pre-cycle 2 LSF median was 6.5% (range, 2.9-16.8%; IQR, 4.2%). Estimated lung doses were <30 gray (Gy) per treatment or <50 Gy cumulative; therefore, no dose reduction was performed in patients with an LSF >10%. RE treatment cycles were performed with resin beads only (three patients), glass beads only (four patients), or alternating resin or glass beads (two patients). Median targeted liver and tumor mass for cycle 1 was 0.57 kg (range, 0.07-2.05 kg; IQR, 1.12 kg) and for cycle 2 was 0.77 kg (range, 0.11-2.08 kg; IQR, 0.94 kg). Radiation doses (Gy) to lungs and perfused liver can be found in Table 2 expressed as medians and ranges with IQR for each treatment cycle. No positive correlation was identified between either radiation dose (Gy) to the perfused liver (cycle 1 rho=0.04, cycle 2 rho=-0.56) or targeted tissue mass (cycle 1 rho=0.04, cycle 2 rho=0.50) and degree of hepatotoxicity/REILD.

**Table 2:** Treatment characteristics related to each radioembolization treatment cycle RE: radioembolization; C1: cycle 1; C2: cycle 2; kg: kilograms; Gy: gray; IQR: interquartile range

Characteristic	Value
Repeat RE territory, n	
unilobar	4
segmental	3
mixed	2
Microsphere type, n	
resin only (C1 and C2)	3
glass only (C1 and C2)	4
resin and glass (mixed cycles)	2
Time interval between treatment cycles in months	
median	9
range; IQR	2-17; 11.3
Lung shunt fraction	
Pre-C1, %	
median	5.5
range; IQR	2.7-10.2; 5.0
Pre-C2, %	
median	6.5
range; IQR	2.9-16.8; 4.2
Targeted liver + tumor mass, kg	
C1	
median	0.57
range; IQR	0.07-2.05; 1.12
C2	
median	0.77
range; IQR	0.11-2.08; 0.94
Radiation dose to perfused liver, Gy	
C1	
median	110.58
range; IQR	35.57-209.33; 114.23
C2	
median	96.58
range; IQR	37.65-219.16; 155.18
Cumulative	
median	213.46
range; IQR	73.22-428.49; 239.86
Radiation dose to lungs, Gy	
C1	
median	3.9
range; IQR	0.04-4.64; 2.14
C2	
median	5.4
range; IQR	1.55-12.13; 6.22
Cumulative	
median	9.99
range; IQR	1.95-16.03; 8.26

Prior, inter-cycle or follow-up period treatments

None of the patients included in the cohort had received prior systemic chemotherapy. Six patients were completely treatment naïve at the time of cycle 1. While three of the six did not undergo intercycle treatment, the remaining three were treated with either overlapping segmental or lobar trans-arterial chemoembolization (TACE) or non-overlapping segmental TACE between cycles 1 and 2. Three patients had undergone treatments prior to cycle 1, including some combination of right hepatectomy, overlapping segmental TACE, overlapping laparoscopic microwave ablation, and/or non-overlapping segmental TACE. Of note, two patients received an additional treatment within the post-cycle 2 follow-up period. One of these patients received a non-overlapping, contralateral RE segmentectomy 49 days post-cycle 2 treatment, and the other received an overlapping lobar TACE 27 days post-cycle 2 treatment. No positive correlation was identified between the number of prior, inter-cycle, and/or follow-up period treatments and the degree of hepatotoxicity (rho=-0.28).

Clinical, imaging and laboratory toxicity assessment

Following cycle 1, 22 objective AE were identified according to CTCAE v.5 (17 grade I, four grade II, and one grade III), with grade I, II, and III AE experienced by 78%, 33%, and 11% of patients, respectively. Additionally, three grade I subjective AE were experienced by 33% of patients following cycle 1. Following cycle 2, 19 objective AE were identified according to CTCAE v.5 (11 grade I, seven grade II, and one grade III), with grade I, II, and III AE experienced by 89%, 56%, and 11% of patients, respectively. Additionally, nine grade I and one grade III subjective AE were identified following cycle 2, experienced by 67% and 11% of patients, respectively. No patient experienced any grade IV or V CTCAE toxicities/AE. Of note, given the median interval of nine months between treatment cycles and multiple patients undergoing inter-cycle treatment, new baseline data was recorded prior to cycle 2 and subsequently used in grading post-cycle 2 AE (Table 3).

**Table 3:** Severity and types of adverse events following each radioembolization treatment cycle according to CTCAE v.5.0 CTCAE v.5.0: National Cancer Institute common terminology criteria for adverse events, version 5.0; AE: adverse events; GERD: gastroesophageal reflux disease * considering new baseline established prior to cycle 2 ^@^ liver enzymes include alkaline phosphatase, aspartate transaminase, alanine transaminase

Subjective adverse events				
Type	Cycle 1		Cycle 2	
	Grade	AE, n (% affected)	Grade	AE, n (% affected)
Abdominal pain	I	1 (11%)	I	4 (44%)
Nausea	I	1 (11%)	I	3 (33%)
			III	1 (11%)
GERD	-	-	I	1 (11%)
Fatigue	I	1 (11%)	I	1 (11%)
Objective adverse events				
Type	Cycle 1		Cycle 2*	
	Grade	AE, n (% affected)	Grade	AE, n (% affected)
Ascites	I	2 (22%)	I	1 (11%)
	III	1 (11%)	III	1 (11%)
Arterial access site pseudoaneurysm	-	-	II	1 (11%)
Pleural effusion	-	-	I	1 (11%)
Hyperbilirubinemia	I	5 (56%)	I	2 (22%)
	II	2 (22%)	II	3 (33%)
Hypoalbuminemia	I	4 (44%)	I	2 (22%)
	II	1 (11%)	II	3 (33%)
Elevated serum creatinine	I	2 (22%)	-	-
Elevated liver enzymes^@^	I	4 (33%)	I	1 (11%)
	II	1 (11%)	II	1 (11%)

At three months post-cycle 1, the median MELD score was 18 (range, 7-24; IQR, 16), and the median Child-Pugh was 6/A (range, 5/A-11/C; IQR, 3) from baseline median of 10 (range, 6-22; IQR, 15) and 6/A (range, 5/A-7/B; IQR, 1), respectively. At three months post-cycle 2, the median MELD score was nine (range, 7-37; IQR, 23), and the median Child-Pugh was 7/B (range, 6/A-9/B; IQR, 2) from baseline medians of nine (range, 7-35; IQR, 16) and 6A (range, 5/A-10/C; IQR, 2), respectively. Following treatment cycle 2, a trend was observed suggesting a positive correlation between higher MELD (rho=0.7) and Child-Pugh (rho=0.74) scores and the degree of post-RE hepatotoxicity/REILD.

**Figure 1:**
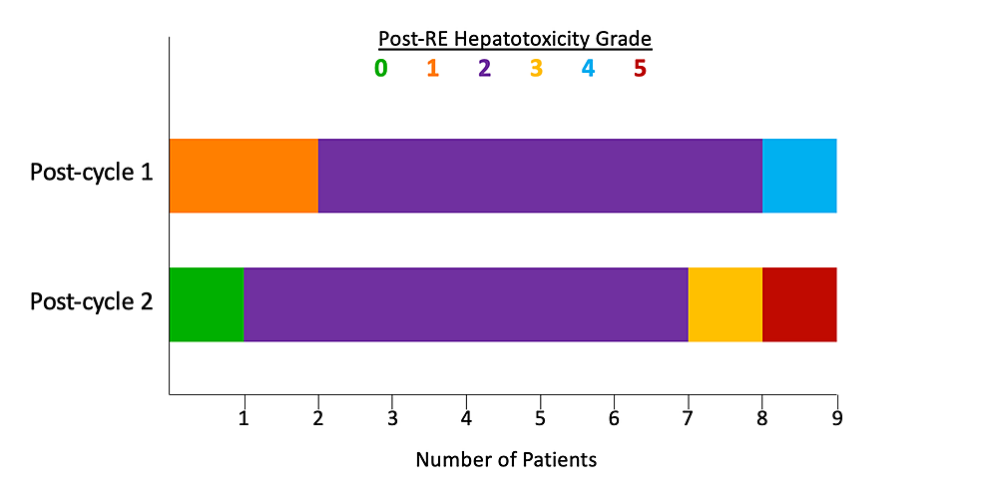
Cohort post-RE hepatotoxicity grade distribution following each treatment cycle RE: radioembolization

When applying the post-RE hepatotoxicity grading system proposed by Braat et al. [3] to this cohort (Figure 1), 100% of patients developed some degree of hepatotoxicity following cycle 1 (grade 1: n=2, 22%; grade 2: n=6, 67%, grade 4: n=1, 11%), and 89% of patients developed some degree of hepatotoxicity following cycle 2 (grade 2: n=6, 67%, grade 3: n=1, 11%, and grade 5: n=1, 11%). When examining individual patient cumulative hepatotoxicity trends, three patients (33%) demonstrated grade progression, five (56%) demonstrated grade stability, and one (11%) demonstrated grade regression. In patients experiencing grade 3+/REILD: one patient progressed from grade 2/moderate hepatotoxicity following cycle 1 to grade 3/REILD (manageable by non-invasive measures) following cycle 2, and one patient developed grade 4/REILD (requiring invasive treatment - paracentesis) following cycle 1, which progressed to grade 5/fatal REILD following cycle 2.

Survival

**Figure 2:**
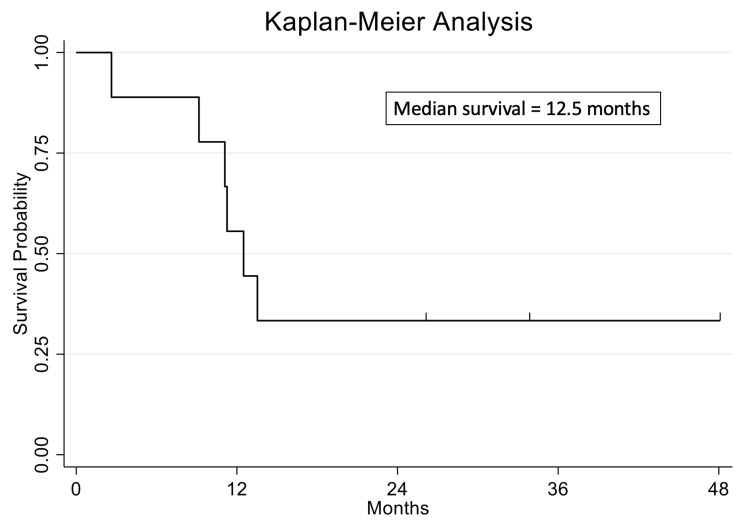
Survival probability following cycle 2 radioembolization Kaplan-Meier graph displaying survival probability after cycle 2 radioembolization. Of note, two patients in the cohort were still alive at the time of analysis.

According to the EMR review, of the nine patients included in this cohort, seven were found to be deceased at the time of writing. The median overall survival following cycle 2 RE was 12.5 months (range, 2.6-69.2 months; IQR, 4.4 months) (Figure 2). At the time of writing, the two surviving patients were at a median interval of 28.9 months (range, 25.1-32.8 months) post-cycle 2 treatment.

## Discussion

Overall, limited data exists detailing the AE/hepatotoxicity profile and incidence of REILD following repeat RE to the same or overlapping vascular territories. Furthermore, comparing the results from previous studies investigating this topic is limited by multiple factors, including the use of varying AE grading systems, non-uniform characterization of the defining features of REILD, and heterogeneity in the study populations with respect to both etiology of malignancy (primary hepatic and/or metastatic neoplasm), and the baseline status of liver function. Table 4 provides a summary of these studies highlighting study characteristics and reported incidence of AE/REILD.

**Table 4:** Summary of AE/REILD incidence in previously published studies following repeat radioembolization to the same or overlapping territories RE: radioembolization; n: number; ICC: intrahepatic cholangiocarcinoma; HCC: hepatocellular carcinoma; CTCAE: National Cancer Institute common terminology criteria for adverse events; AE: adverse events; REILD: radioembolization induced liver disease ^a ^defined by study authors in patients undergoing repeat RE to same or overlapping distributions

Reference	Total, n	Repeat RE, n	Baseline liver dysfunction or cirrhosis, n	Malignancy type, n	AE grading system	Grade 1-2 AE	Grade 3+ AE	REILD^a ^n, (%)	Fatal REILD^a ^n, (%)
pre-Sangro et al. [11]									
Goin et al. [9]	88	12	generally present in patient cohort	HCC, 12	Southwest Oncology Group	not reported	4	n/a	n/a
Young et al. [13]	41	41	30	HCC, 41	none	n/a	n/a	n/a	n/a
post-Sangro et al. [11]									
Lam et al. [4]	247	8	heavily pretreated/ salvage patients	secondary malignancy, 8	CTCAE v4.02	8	2	2 (25)	2 (25)
Zarva et al. [10]	21	21	0	HCC, 8; secondary malignancy, 13	CTCAE v4.02	125	3	0 (0)	0 (0)
Filippi et al. [14]	9	9	not specified	recurrent ICC, 9	CTCAE v4.02	9	0	0 (0)	0 (0)
Elsayed et al. [8]	39	39	17	HCC, 17; secondary malignancy, 22	CTCAE v5	40	3	3 (7.7)	0 (0)
Masthoff et al. [17]	68	11	not specified; 9 of 11 pretreated	HCC, 3; ICC, 3; secondary malignancy, 5	none	n/a	n/a	0 (0)	0 (0)

In this study's very specific cohort of patients with HCC in the setting of background cirrhosis (i.e., limited hepatic reserve and/or decreased baseline liver function) treated with two cycles of RE to the same or overlapping vascular distributions, the resulting CTCAE AE/toxicities shared a mix of findings detailed in prior studies focused on repeat RE. Nearly all of the objective AE seen in this study were grade I or II (Table 3), similar to results reported by Elsayed et al. [8], Zarva et al. [10], and Fillippi et al. [14]. A single patient, who ultimately experienced fatal REILD, developed grade III symptomatic ascites requiring paracentesis following both cycle 1 and cycle 2. Furthermore, 24-25% of the grade I/II post-cycle 1 and 14-28% of the grade I/II post-cycle 2 AE were found to be transient and self-limited. Like those previously reported in the literature, the observed categories of AE/toxicities included elevated liver enzymes (serum aspartate aminotransferase [AST], alanine transaminase [ALT], alkaline phosphatase), hypoalbuminemia, hyperbilirubinemia, and ascites. No CTCAE grade IV or V AE were identified. Of note, in addition to experiencing limited hepatic functional reserve/decreased baseline liver function because of cirrhosis, six of the nine patients were treated with at least one and as many as six additional locoregional or surgical interventions prior to, between, and/or within the post-cycle 2 follow up period, which almost certainly further limited hepatic functional reserve.

Due to the previously discussed lack of a consensus REILD definition, it is difficult to directly compare the incidence of REILD in this cohort to that described in the literature. Accordingly, the REILD definition and post-RE hepatotoxicity grading scale put forth by Braat et al. [3] were applied, resulting in 100% and 89% of patients experiencing a varying degree of post-RE hepatotoxicity following treatment cycle 1 and 2, respectively. By definition, grades 3-5 are indicative of REILD, ranging in severity from that which can be managed by non-invasive means to fatal REILD. Following cycle 1, a single patient developed REILD (grade 4), which progressed to fatal REILD (grade 5) following cycle 2. Following cycle 2, an additional patient advanced from grade 2 post-RE hepatotoxicity to grade 3 REILD.

Many risk factors for developing post-RE hepatotoxicity/REILD have been described, including prior RE [3], other previous intra-arterial therapies [7], the number of prior liver-directed therapies [15], administered activity per target volume [11], liver volume [2], and decreased functional hepatic reserve/baseline liver function [2,16].

The personalized versus standard dosimetry approach of selective internal radiation therapy in patients with locally advanced hepatocellular carcinoma (DOSISPHERE-01) trial recently brought personalized dosimetry to the forefront of radioembolization-related research. In that randomized, multicenter, investigator-sponsored phase II trial comparing the clinical outcomes of patients with intermediate/advanced HCC using two pre-treatment dosimetry determination methods: (1) standard, single-compartment dosimetry (STD), or (2) personalized dosimetry (PERSO) [20]. Salient findings from the DOSISPHERE-01 trial included an increased overall response rate of 50% in the PERSO arm versus 14.3% in the STD arm and an acceptable and comparable safety profile between the two groups, even accounting for the fact that treatment was intensified for 75% of patients in the PESRO arm. Clinically relevant REILD occurred in 8.9% of treated patients who received standard and personalized dosimetry. However, as opposed to our study's cohort, patients were selected for the DOSISPHERE-01 trial only if they had good liver function and limited spread of liver disease with the possibility of sparing at least 30% of the liver from radiation [20].

Interestingly, in this cohort, no positive correlations between radiation dose (Gy), targeted tissue mass, or the number of prior or inter-cycle treatments and post-RE hepatotoxicity/REILD were identified. However, following treatment cycle 2, trends were observed suggesting a positive correlation between higher MELD and Child-Pugh scores and the degree of post-RE hepatotoxicity. Given that all patients had inherently limited hepatic functional reserve/baseline liver dysfunction secondary to cirrhosis, MELD and Child-Pugh scores may have a role in predicting the risk of post-RE hepatotoxicity. Although this study's limited sample size prevents drawing such robust conclusions from these data alone, following RE, others have reported higher MELD scores associated with a greater incidence of grade II or greater AE [8] and decreasing survival with increasing severity of Child-Pugh disease [16].

Limitations of this study most notably include its single-center retrospective design, limited sample size, and cohort treatment history inhomogeneity. While the retrospective design potentially allowed for consideration of how multiple prior treatments may correlate with worsening hepatotoxicity, associations related to repeat RE in isolation were unable to be derived. Furthermore, prior, inter-cycle, and/or follow-up period treatments were of mixed type and may or may not have involved the same target distribution as the RE treatments detailed in the current study. While these additional treatments may be considered confounding factors, the lack of a positive correlation associated with post-RE hepatotoxicity in a patient cohort with already decreased baseline functional hepatic reserve may be noteworthy.

## Conclusions

In conclusion, post-RE hepatotoxicity following repeat RE to the same or overlapping vascular territories in patients with limited functional reserve/baseline liver dysfunction is a common occurrence with variable severity ranging from transient laboratory derangement to fatal REILD. While this study's sample size prohibits strong association statements, MELD and Child-Pugh scores may serve as predictors of post-RE hepatotoxicity incidence and/or severity which supports similar statements in the literature. Furthermore, after an extensive literature review, it seems readily apparent that the current lack of a consensus REILD definition and grading scale results in non-uniform reporting of incidence as well as clinical and laboratory features of the disease process. Additionally, without an independent entry under the CTCAE, it is difficult to accurately characterize post-RE specific hepatotoxicity other than by parts of the whole. Strides aimed at improving clinical characterization, forming a more complete diagnostic definition, and establishing a uniform grading system with respect to REILD are of particular importance and would ultimately improve repeat RE patient selection and risk management.
